# Past, present and future of IOA from my eyes and mind

**DOI:** 10.4103/0019-5413.43408

**Published:** 2008

**Authors:** HKT Raza

**Affiliations:** *Professor and Head of Orthopaedics, NSCB Medical College, Jabalpur, MP, India*

Honorable members of the Indian Orthopaedic Association (IOA), ladies and gentlemen.

As I come to the end of this very gratifying period of my life, I wish to take you to the journey of the Association. It was during the Conference held in 1996 that you chose Jabalpur to host the 1998 annual Conference (IOACON). It is a matter of providence that I am also remitting the office of President of the Association at Kolkata.

Buoyed by the success of the Jabalpur conference, you encouraged me to contest for and elected me to the post of Honorary Secretary of IOA (1999). The last decade has been a passionate love affair with the IOA for me.

A Presidential Address is supposed to be a reflection of the past of the organization, stock taking of the current achievements and a direction for the future. This address has therefore been divided into three parts: past, present and future of IOA from my eyes and mind.

## PAST

I bow down in reverence to Dr. B. N. Sinha, Dr. B. Mukhopadhya and 12 other visionaries for starting the Orthopaedic Section of the Association of Surgeons of India (A.S.I.). They nurtured the association in its formative years. A number of giants have subsequently occupied the chair of the President of the Association, guiding its destiny. Some important landmarks in our association have been:

Declaration of starting an Orthopaedic Section of the A.S.I. at Hyderabad (1954), first Conference of the Section at Amritsar (1955), Kini Memorial Oration (1958), Johnson and Johnson Fellowship (1959), publication of I.J.O (1967) and the first ratification of the IOA Constitution (1967). IOA held the Silver Jubilee Conference at Bombay (1980), where the CME programme was started. The Silver Jubilee commemoration lecture was started at Varanasi (1982). The resolution to separate from A.S.I. was passed in (1986) at IOACON, Cuttack, and ratified in the December 1986 meeting of the A.S.I. at Agra.

The subsequent two decades between 1980 and 1999 saw a consolidation of activities of the Association into three major categories:

1. Staging of the annual conference.
2. Publication of the journal.
3. Instructional course lectures of IOA (1995).

IOA had a constitution but no protocols or guidelines for day-to-day functioning and for the manner in which conferences/other activities of the Association were to be organized. Much of the decision making was left to the discretion of the President/Honorary Secretary of the IOA with some input from the Executive Committee. The organizing secretaries were not given any guidelines for the conduct of the conferences. The end result was some excellent conferences and some frightfully badly organized ones. There was no coordination between the state chapters and the parent body.

## TURN OF THE MILLENIUM (PRESENT)

The period between 2000 and 2006 can certainly be called a watershed in the history of the IOA We worked on all fronts. On the academic front, postgraduate (PG) education was given a constant attention and the national PG quiz was started and strengthened. We brought a constitutional amendment to improve the working of the association along with establishing guidelines for various scientific activities such as annual conferences, CMEs and pre-conference workshops. The affiliation of state chapters was started to give the IOA a democratic look and the member directory was published. The IOA website and a regular publication of the newsletter was started to communicate better with the members. Lots of fellowships and opportunities were created for training the younger generation. To improve the scientific deliberation, many gold medal awards were initiated to recognize the talent and stimulate budding orthopaedic surgeons to conduct research. The national policy of disaster management and orthopaedic training methodology for orthopaedic teachers were initiated. These will pay dividends in years to come.

The “PPRP–Free India Campaign” was launched during the Golden Jubilee conference in which nearly 20,000 operations were performed nationwide by our members for disabled persons, especially those for the polio affected. It gives me immense satisfaction that my theme for 2007, “Focus on the Golden Hour” has been taken up by members all over the country. We have undertaken public awareness campaigns by distributing CDs on first aid to trauma victims especially developed to educate the common masses. The lectures, rallies, symposia workshops, lectures in state conferences, articles in the local press and TV talks were held throughout the year. The text book on emergency care was released. Advanced trauma life support and basic trauma life support courses were conducted. The theme has therefore more than served its purpose.

## FUTURE

There should be a continuity between the present and the future for any organization to progress. The achievements of today have to be consolidated for the sustained growth of our association.

### International presence

India has world-class hospitals, competent consultants and a treasure of clinical material. The time is ripe for us to showcase our enormous experience and talent on the world stage. We are holding the Asia Pacific Orthopaedic Association (APOA) Congress in 2012, in its Golden Jubilee year. We wish that the APOA 2012 Congress at New Delhi showcases Indian Orthopaedics. May I request all of you to take up specific projects, document your cases well, follow world literature on the topics during this time and come up with excellent scientific papers for the APOA Congress.

We have been able to open up fruitful dialogues for cooperation between the IOA and other national organizations such as the Australian Orthopaedic Association, the British Orthopaedic Association, the Indian Orthopaedic Society (UK), the Pakistan Orthopaedic Association and the Bangladesh Orthopaedic Association. We need to play a proactive role in nurturing orthopaedic services and training in SAARC countries as there is a vast similarity in clinical material, patient profile and infrastructure, and, therefore, in solutions to the clinical problems as well.

We boast of a wealth of experience from quality orthopaedic care. Unless we publish, much of our work will be hidden from the global eyes. Hence, we need to create an attitude toward publication. Printing of the Journal of Bone and Joint Surgery (JBJS, British) in India will allow our members to procure subsidized JBJS for upgrading their knowledge.

### Orthopaedic education in India

The emphasis on education is the best investment of the future. With growing industrialization, increased number of automobiles and poor roads, there is an increase in the incidence of trauma. With an increase in life expectancy, there will be an ever increasing geriatric population. This will increase the burden of degenerative musculoskeletal problems and osteoporosis. A bulk of the initial consultation/medical help will be borne, as always, by general practitioners and doctors posted in the periphery. There is therefore a need for a proper training of the undergraduate students in orthopaedics. Training without proper evaluation that is not effectively represented in results has no value. Across the country, we are noticing a paradigm shift of student interest away from orthopaedic clinical postings, lectures and evening clinics as there is no separate evaluation of orthopaedics in the undergraduate curriculum. The M.C.I. still deems it fit to club orthopaedics with surgery. We have represented this problem to the M.C.I. on several occasions, but to no avail. I feel a concerted effort is needed to make it a separate subject. At the same time, we must device mechanisms to make our delivery to the undergraduates more interesting so that not only the basic orthopaedic care delivery improves but also the best of merit joins orthopaedics as a PG student.

#### Postgraduate training

A beginning has been made by the initiation of a dialogue among teachers concerned with the disparity of PG teaching in India. A white paper is being read on this topic in 2007. We are in the process of bringing out a revised standard Orthopaedic curriculum for PGs, a book on quality control and a standard log book. It is hoped that this will help in streamlining the PG training. The IOA has recently also organized the first national seminar on Training Methodology for our young orthopaedic faculty members. Such annual activities will motivate them to contribute their talent to teach and train undergraduates and PGs.

#### Post qualification training

In the fast developing medical world, it is essential to keep oneself abreast with recent developments. The state chapters and city clubs have been encouraged to organize regular academic events like conferences, workshops and seminars. This has helped our fraternity enormously. Still, we need to create a structured network of such avenues where knowledge can be upgraded. The available fellowships (inland and overseas) need to be properly advertised. I appeal to our senior colleagues to provide more inland/overseas fellowships. We also need to identify various Centers of Excellence in public and private sectors for short-term training in various subspecialties.

### Annual conferences

Streamlining of protocols and guidelines for IOACONs has helped us maintain excellent organizational standards. We have much better organized conferences now than in the past despite the evergrowing number of participants. If the scientific programme is finalized well in advance, it is printed in time and mailed to members at least 1 month before the conference such that members can plan to attend programmes/lectures/sessions of their interest and derive maximum benefit. Our annual instructional course lecture programme needs a revamp so that it has more interactive sessions.

### Public service activities

Every academic organization needs to have activities directly benefitting the society. We need to develop public awareness campaigns to educate the society. An effort has already been made by the IOA during 2007 by educating the common masses about the golden hour in trauma. We need to talk on more of such issues that directly affect the general health, for example “concept of bone health,” osteoporosis (Prof. S. C. Goel's theme) and many more. Future presidents should select themes that are of practical significance.

### Indian Journal of Orthopaedics (IJO)

The journal has certainly shown remarkable improvement in both the quality of production as well as its content. The IOA has a vision for the IJO We can extend the core support to the journal by our good quality articles and state of the art peer review. I call upon all of you to be active in both the fronts to make it a journal of international repute.

### Publication of text books by our members

I am glad to inform you that the second edition of the IOA text book of Trauma and Orthopaedics has been released. More and more text writing by our authors will be a guide source material for treatment of orthopaedic ailments in our milieu.

### Research in orthopaedics in India

There is very little original research in the field of orthopaedics in India. From my own experience on several original projects, I personally feel that motivation and aptitude is more important than the availability of resources. One does not require large amounts of money for every research project, e.g. a lot of anthropometric work can be carried out without much funding. Good work done by lots of our colleges will inspire our young orthopods to indulge in this rewarding activity. We must develop technology and implants suited to the needs of our patients. Search for cost-effective treatment protocols will always remain a vast area for research in this part of the world.

### Association

All state chapters should get an affiliation to make our voice strong. Democracy works best if we vote; hence, we should develop a mechanism that most of the members vote. We shall rise above the regional sentiment during the election to select the best candidate to serve this august body. The IOA benevolent fund and the IOA professional indemnity scheme are developed for the benefit of our members and hence we should be utilizing them.

I am looking forward to handing over the baton to my worthy successor Prof. S. C. Goel with the assurance that I shall render my services whenever the IOA requires it.

Before I conclude, I must thank all members, all past presidents and all members of the executive committees between 2000 and 2007 for all the love, help and cooperation that they have given me.

I thank my mother Mrs. Roshan Raza, my wife Kehkashan, my daughter Falaq Zehra and my son Shamikh for suffering so much by my involvement with IOA but always remaining exceptionally supportive

JAI HIND

JAI IOA

